# Supporting self-managed abortion care in “practice not premise”: a qualitative study of provider perspectives, roles, and information pathways to care in India*

**DOI:** 10.1080/26410397.2025.2531680

**Published:** 2025-07-21

**Authors:** Laura E. Jacobson, Caila Brander, Balasubramanian Palanisamy, Sruthi Chandrasekaran, Blair G. Darney, Julia M. Goodman, Ruvani Jayaweera, Caitlin Gerdts

**Affiliations:** a Assistant Professor, Portland State University, OHSU-PSU School of Public Health, Portland, OR, USA; Independent Consultant, Ibis Reproductive Health, Oakland, CA, USA.; b Senior Research Manager, Ibis Reproductive Health, Oakland, CA, USA; c Executive Director, Rural Women’s Social Education Centers (RUWSEC), Tamil Nadu, India; d Independent Consultant, Ibis Reproductive Health, Oakland, CA, USA; e Associate Professor, OHSU-PSU School of Public Health, Portland, OR, USA; Associate Professor, Oregon Health and Science University, Portland, OR, USA; Affiliate Professor, Instituto Nacional de Salud Publica (INSP), Centro de Investigacion en Salud Poblacional (CISP), Cuernavaca, Mexico; f Associate Professor, OHSU-PSU School of Public Health, Portland, OR, USA; g Senior Research Scientist, Ibis Reproductive Health, Oakland, CA, USA; h Vice President of Research, Ibis Reproductive Health, Oakland, CA, USA

**Keywords:** abortion, self-managed abortion, pharmacy, quality of care, India

## Abstract

This qualitative study explored provider perspectives on self-managed abortion (SMA) in India, their roles, and how they share information about pathways to both clinician- and self-managed abortion care. We conducted 33 semi-structured interviews with a range of providers (medical, community health, and pharmacy) in three states in India: Jharkhand, Bihar, and Tamil Nadu. Using thematic analysis, we examined provider perspectives on SMA, their involvement in abortion care, and how they contribute to information sharing around access pathways. We categorised findings by provider type, direction of care pathways, abortion modality (clinician-managed vs. SMA), and the kind of care delivered. Our findings showed most providers described abortion as conditionally acceptable and primarily encouraged clinician-managed care. Concerns about SMA safety and potential liability often led them to discourage SMA. Nonetheless, participants acknowledged three areas where providers played a role in SMA: providing information, dispensing medication, and providing support (i.e. managing pain). Pharmacy workers and local providers shared information with abortion seekers on pathways to access SMA care. Some community health workers directed clients to pharmacies, but more often only provided SMA information and support. Despite provider concerns, support for and pathways to SMA exist in India. Understanding the dynamics of provider perspectives and roles can inform improvements to comprehensive reproductive health policies and programmes in order to promote person-centred abortion care – including SMA – and address provider concerns. Synergies are needed between the formal health sector and SMA support networks to advance person experiences and reinforce quality abortion care as a human right.

## Plain language summary

This study explored how various healthcare providers in India perceive self-managed abortion (SMA). It also looked at how they provide information about both abortion by clinicians and SMA. We found that most providers accept abortion on some conditions. They generally recommend abortion by clinician rather than SMA. They have concerns about SMA safety and potential liability for themselves. Despite these concerns, providers still play supportive roles in SMA. These include offering information, dispensing medications, and helping with issues like managing pain. Pharmacy workers and local health providers often guide abortion seekers towards self-managed care options. Community health workers tend to direct individuals to pharmacies for SMA resources and support. The findings show that support structures and informational pathways exist. Integrating these into broader reproductive health policies could enhance person-centred abortion care. Addressing the concerns of providers would promote safer and more accessible abortion care. Understanding provider perspectives and roles in SMA can help to improve comprehensive reproductive health programmes. Increased collaboration between formal health services and community-based SMA support networks is needed.

## Résumé

Cette étude qualitative a exploré le point de vue des prestataires sur l’avortement autogéré en Inde, leurs rôles et la manière dont ils partagent les informations sur les parcours d’accès aux soins d’avortement, qu’ils soient pratiqués par un clinicien ou autogérés. Nous avons mené 33 entretiens semi-structurés avec divers prestataires (médicaux, de santé communautaire et pharmaceutiques) dans trois États indiens: le Jharkhand, le Bihar et le Tamil Nadu. Avec une analyse thématique, nous avons examiné le point de vue des prestataires sur l’avortement autogéré, leur participation aux soins d’avortement et leur contribution au partage d’information sur les parcours d’accès. Nous avons classé les résultats par type de prestataire, orientation des parcours de soins, modalité d’avortement (clinique ou autogéré) et type de soins prodigués. Nos résultats ont montré que la plupart des prestataires décrivaient l’avortement comme acceptable sous certaines conditions et encourageaient principalement les soins cliniques. Les inquiétudes concernant la sécurité de l’avortement autogéré et sa responsabilité potentielle les incitaient souvent à le déconseiller. Néanmoins, les participants ont dégagé trois domaines où les prestataires avaient joué un rôle dans l’avortement autogéré: l’information, la délivrance de médicaments et le soutien (c’est-à-dire la gestion de la douleur). Les pharmaciens et les prestataires locaux ont partagé des informations avec les personnes souhaitant avorter sur les voies d’accès aux soins pour un avortement autogéré. Certains agents de santé communautaires ont orienté les patientes vers les pharmacies, mais se sont le plus souvent limités à fournir des informations et un soutien concernant l’avortement autogéré. En dépit des préoccupations des prestataires, un soutien et des voies d’accès à l’avortement autogéré existent en Inde. Comprendre la dynamique des points de vue et des rôles des prestataires peut contribuer à améliorer les politiques et programmes complets de santé reproductive afin de promouvoir des soins d’avortement centrés sur la personne, y compris l’avortement autogéré, et de répondre aux préoccupations des prestataires. Des synergies sont nécessaires entre le secteur de la santé formel et les réseaux de soutien à l’avortement autogéré afin d’améliorer l’expérience des personnes et de renforcer la qualité des soins d’avortement en tant que droit humain.

## Resumen

Este estudio cualitativo exploró las perspectivas de los prestadores de servicios sobre la autogestión del aborto (AGA) en India, sus roles y cómo comparten información sobre las rutas tanto a los servicios de aborto ofrecidos por profesionales médicos como a la autogestión del aborto. Realizamos 33 entrevistas semiestructuradas con una variedad de prestadores de servicios (médicos, salud comunitaria y farmacias) en tres estados en India: Jharkhand, Bihar y Tamil Nadu. Utilizando análisis temático, examinamos las perspectivas de los prestadores de servicios sobre la AGA, su participación en los servicios de aborto y cómo ellos contribuyen al intercambio de información sobre el acceso a las rutas. Categorizamos los hallazgos por tipo de prestador de servicios, dirección de las rutas a la atención, modalidad de aborto (gestionado por médicos vs. autogestionado), y tipo de atención brindada. Nuestros hallazgos mostraron que la mayoría de los prestadores de servicios describieron el aborto como condicionalmente aceptable y, en su mayoría, recomendaron la atención gestionada por profesionales médicos. Las preocupaciones sobre la seguridad y el posible riesgo de la AGA a menudo les llevaban a desalentar la AGA. Sin embargo, los participantes reconocieron tres áreas donde los prestadores de servicios desempeñaron un papel en la AGA: proporcionando información, dispensando medicamentos y brindando apoyo (ej., manejo del dolor). Los trabajadores de farmacias y prestadores de servicios locales compartieron con las personas que buscaban un aborto información sobre las rutas para acceder a la AGA. Algunos trabajadores en salud comunitaria dirigían a sus pacientes a farmacias, pero con mayor frecuencia solo proporcionaban información sobre la AGA y apoyo. A pesar de las preocupaciones de los prestadores de servicios, existen en India apoyo y rutas a la AGA. Entender las dinámicas de las perspectivas y funciones de los prestadores de servicios puede incidir en mejorar políticas y programas de atención integral a la salud reproductiva a fin de promover servicios de aborto centrados en cada persona, incluida la AGA, y abordar las preocupaciones de los prestadores de servicios. Se necesitan sinergias entre el sector salud formal y las redes de apoyo a la AGA para avanzar las experiencias de las personas y reafirmar los servicios de aborto de calidad como un derecho humano.

## Introduction

In India, the Medical Termination of Pregnancy (MTP) Act of 1971 has permitted legal abortions to save a pregnant person's life, to protect physical and mental health in instances of economic and social need, for fetal abnormalities, and in cases of contraceptive failure for married couples.^1^ This policy was also recently amended to allow care up to 24 weeks of gestation under certain conditions.^2^ However, despite liberal abortion laws, equitable access to high-quality abortion care remains a challenge.^3^ Access to safe abortion is widely recognised as fundamental to reproductive autonomy, bodily integrity, and gender equity where international law and frameworks affirm that individuals have the right to make decisions about pregnancy and to access necessary healthcare without discrimination or undue barriers.^4–6^

Reflecting this rights-based approach, the World Health Organization (WHO) has increasingly embraced the expansion of abortion access and recommends abortion providers include a broad array of health workers including non-clinically trained community health and pharmacy workers.^7^ However, in India the provision of abortion services is restricted to registered physicians in authorised clinics.^1^ Despite this policy, data from 2015 show that only 22% of the estimated 15.6 million abortions that occur annually in India took place in registered health facilities, whereas 73% of all abortions were medical abortions that took place outside of health facilities.^8^

Self-managed medical abortion (SMA) has been shown to be safe and effective,^9–13^ and has recently been incorporated into the WHO’s abortion care guidelines as a recommended model of abortion care for <12 weeks of gestation.^6^ WHO guidelines stipulate that SMA should be accompanied with accurate information, quality-assured medications, and access to trained health workers and health system referral services if needed or wanted.^6^ Self-care health interventions like SMA are seen as strategies to improve universal health coverage by reaching underserved populations, and WHO’s abortion care guidelines are explicitly underpinned by human rights, ethics, and gender equity.^6^ SMA occurs on a continuum where people interact with health workers to varying degrees throughout the process, including some interactions with community health workers or direct pharmacy-facilitated care.^14,15^ Previous work has documented that while SMA is prevalent in India,^8^ gaps exist in consistently available high quality information sources from pharmacies.^16^

Currently, there is little evidence globally about provider perspectives on SMA, the roles they play, or how the health system and healthcare personnel interact with people seeking or obtaining SMA. A key innovation of this study is the focus on interactions between the health system and SMA practices. The purpose of this study is to explore perspectives on SMA from a diverse group of providers (medical, community and pharmacy); understand the roles they play in SMA; and describe provider information sharing on pathways to clinician- and self-managed abortion care in India.

## Methods

We conducted a qualitative analysis of 33 semi-structured interviews with a diverse range of providers (medical, community health, and pharmacy) in India. This is a sub-analysis of a larger qualitative study focused on SMA experiences, pathways to care, and support networks in India. The findings of the larger study are reported elsewhere.^17^ Briefly, the larger study conducted qualitative interviews and focus group discussions with various stakeholders, including women of reproductive age, medical abortion users, their partners and families, and providers to understand the medical abortion user journey and preferences around accessing information on medical abortion. The sample of healthcare providers in this sub-analysis included Bachelor of Medicine, Bachelor of Surgery (MBBS)-qualified doctors providing abortion care; community health workers (informal local providers, accredited social health activists (ASHAs), Anganwadi workers, health counsellors, and auxiliary nurse midwives); and pharmacy workers (pharmacists, chemists, and shop workers). MBBS-qualified doctors in this study are medical doctors with credentials recognised by state and national licensing boards and are generally regarded as having authoritative roles in the health system.^18^ Many MBBS-qualified doctors in this study are Registered Medical Providers (RMPs) based on their roles in obstetric and gynaecological care and are authorised to provide abortion care by the MTP Act^2^; however, we cannot confirm this for all providers so have referred to them as MBBS-qualified doctors. ASHAs and Anganwadi workers are community health workers who function primarily within the public sector to deliver basic health services including sexual and reproductive health care and maternal and child health.^19,20^ Health counsellors are similar to ASHAs and Anganwadi workers but also work in the private health sector. Auxiliary nurse midwives have basic nursing skills and midwifery competencies^21^; and informal local providers (commonly referred to as *Jhola Chaaps* in Jharkhand and Bihar) provide basic health care in communities but operate privately and lack national standards of training, qualifications or authority to write official prescriptions.^19,22^ Pharmacy workers are responsible for dispensing medications and work in both public and private sectors. Recognising that the MTP Act allows licensed abortion providers to prescribe medical abortion pills (up to 7 weeks) from private settings including their clinics and where consultation or follow-up care may occur via telehealth^23^ where abortion pills may be taken under the supervision of a provider but outside of a formal heath facility,^1^ we define clinician-managed abortion as any abortion occurring under the supervision of an MBBS-qualified doctor.

Provider interviewers took place between 3 February 2022 and 8 June 2022 in three states in India: Jharkhand, Bihar, and Tamil Nadu, which represent a range of socioeconomic and health system conditions (see Supplemental Figure S1 for map). Bihar and Jharkhand have the top two highest proportions of households living in poverty in India and have high maternal mortality rates of 164 and 78 deaths per 100,000 live births, respectively,^24,25^ above the global target of <70 per 100,000 live births.^26^ Tamil Nadu has strong public health infrastructure; below the national average proportions of households living in poverty; and one of the lowest maternal mortality rates (56 deaths per 100,000 live births) in India.^24,25,27^

Trained research assistants recruited participants by telephone and social media. Eligible participants were (1) 18 years or older; (2) able to give informed consent; (3) able to speak English/Hindi/Tamil/dialects of these languages; and (4) met at least one of the following criteria: worked as a community health worker in rural areas; worked as a provider in medical facilities licensed to provide abortions; worked as an informal local provider in a community where they may be the first point of contact for health-related concerns; or worked as a chemist, pharmacist, or pharmacy shop worker. Participants were purposively selected based on their knowledge or role in provision of abortion care in their communities.

The study team developed semi-structured interview guides based on the literature review and known gaps in the understanding of abortion medication self-use in India.^8,21^ All participants provided informed consent prior to starting the interview and interviews were conducted by trained research assistants in the local languages. The focus of the provider interviews was to assess the role of a diverse range of providers in abortion care in the community (see Supplemental Figure S2 for the interview guide). The interviews were audio recorded and assigned ID numbers to de-identify the data collected. De-identified interviews were translated and transcribed into English by a professional service. This study received approval from the Institutional Review Board (IRB) of Sigma Research and Consulting, a national IRB in New Delhi, India (IRB number 10034/IRB/21-22) on 30 September 2021.

### Reflexivity statement

The research questions for this study emerged from a stakeholder meeting focused on identifying gaps in abortion research in India. India-based stakeholders emphasised the importance of understanding the perspectives of self-managed abortion (SMA) users, which shaped the study's aims. The lead author, LJ, a white, cisgender doctoral candidate based in the US, conducted this sub-analysis in partnership with Ibis Reproductive Health. Co-authors BGD, JMG, and CG – senior US-based researchers – served as academic advisors on the dissertation. SC, co-principal investigator from India with extensive experience in sexual and reproductive health, and BP, also based in India, oversaw this research and contributed to contextual interpretation of findings. CB, RJ, and CG, affiliated with Ibis, led the broader project and secured funding. The authorship team represents a mix of geographies (India and the USA), disciplines, and career stages, with intentional collaboration across contexts to support locally relevant research practices.

### Analysis

To understand and describe provider perspectives on SMA, their roles, and information sharing on pathways to abortion care in in India, we conducted a qualitative thematic analysis. Two coders (LJ and CB) used a deductive and inductive approach wherein deductive codes were created *a priori* based on gaps in the literature on SMA with a focus on India^8,28^; quality of care frameworks^29,30^ and the study interview guides. To validate the codebook, LJ and CB documented themes and patterns that emerged inductively^31,32^ and met regularly to discuss and modify the codebook. Transcripts were then coded with the modified codes. The final codebook is included in Supplemental Table S1. Next, LJ drafted code summaries on key codes or themes; established links between the study objectives and the summary findings; and developed conclusions from the underlying themes that were evident in the data.^32^ Illustrative quotes of the themes are presented with the type of provider, their number of years in practice, and state of residency.

To describe information sharing from providers on pathways to abortion care, a “pathways” code was applied any time an interviewee mentioned providing information directing an abortion seeker to another provider to access abortion-related care, and a memo was written to describe the interaction. For example, if a person came to a pharmacy to access abortion medications and the pharmacy worker directed them to an MBBS-qualified doctor, this was coded as “pathways” in the direction of pharmacy to clinician-managed abortion care. Next, LJ consolidated these memos and created a figure that included the types of providers, the abortion care modality (clinician- or self-managed), the direction of the pathway to care, and the type of care delivered. All documents were coded in MAX QDA qualitative analysis software (VERBI 2022).

## Results

In total, we conducted 33 provider interviews with: pharmacy workers (*n* = 7, 21.2%); informal local providers (*n* = 6, 18.2%); ASHAs (*n* = 7, 21.2%); Anganwadi worker (*n* = 2, 6.1%); health counsellor (*n* = 1, 3.0%); auxiliary nurse midwives (*n* = 6, 18.2%) and MBBS-qualified doctors (*n* = 4, 12.1%). Providers had a mean age of 36.5 years (range 21–55), and the majority were female (60.6%) with at least a college degree/diploma/technical school education (72.7%). The average years of provider experience was 12.1 (range 2–28 years) (Table 1).

**Table 1.** Participant characteristics, healthcare providers in three states in India: Bihar, Jharkhand, and Tamil Nadu, 2022 (*n* = 33)

Characteristics	Median (range)
**Age**	36 (21–55)
**Years in practice**	11 (2–26)
**Education**	***n* (%)**
Completed secondary	9 (27.3)
College degree/diploma/technical school	17 (51.5)
Postgraduate degree	7 (21.2)
**Married**	29 (87.9)
**Sex**	
Female	20 (60.6)
Male	13 (39.4)
**Religion**	
Islam	5 (15.2)
Hindu	26 (78.8)
Christianity	2 (6)
**Provider type**	
Pharmacy workers (pharmacists, chemists, shop workers)	7 (21.2)
Informal local providers	6 (18.2)
Accredited social health activists (ASHAs)	7 (21.2)
Anganwadi workers	2 (6.1)
Health counsellors	1 (3.0)
Auxiliary nurse midwives	6 (18.2)
MBBS-qualified doctors	4 (12.1)
**State**	** **
Bihar	12 (36.4)
Jharkhand	15 (45.5)
Tamil Nadu	6 (18.2)

Four key themes emerged from these interviews: (1) providers conditionally support abortion care for the “right reason”; (2) providers support SMA in “practice not premise”; (3) providers prefer to direct clients towards clinician-managed abortion over SMA and (4) providers share information on pathways to SMA-related care despite their concerns.

### Providers conditionally support abortion care for the “right reason”

Overall, most providers interviewed for this study did not support abortion (clinician-managed or SMA) under most circumstances. However, several noted that abortion was conditionally acceptable for the “right reason”. This theme was consistent across provider types and these reasons generally related to age, marital status, socioeconomic status, birth spacing, and/or parity. Often providers would say, “if it is the first child, continue with the pregnancy”. Some providers felt that it was acceptable for older, poorer, married women to have an abortion, while others indicated that married women should always continue a pregnancy, but abortion was acceptable for the young and unmarried. One informal local provider talked about economic conditions as a reason to encourage or dissuade abortion:

*“If they earn a good wage then we suggest to them not go for an abortion. If we see that the economic conditions are not good, then we suggest they go for an abortion. And if the economic condition is good and they can nurture the baby properly then we suggest they do not go for an abortion.”* (Informal local provider, 7 years in practice, Jharkhand)

### Providers support SMA in “practice not premise”

When asked about their perspectives on people who self-manage abortions in the community, providers of all types indicated that they do not support the premise of SMA and perceive it to be dangerous for clients. Many respondents described SMA as “a careless act” and stated, erroneously, that it could cause physical complications, including “very rare chances of conceiving again” and serious harm. For these reasons, many believed that abortion “should always be done with a doctor”. However, some providers noted that compared to clinician-managed care, medical abortion accessed directly from pharmacies was the “cheaper option” and they understood it to be more private and accessible. As one ASHA explained:

*“They think easy option is taking the medicine. They think this is less expensive and they don't have to go anywhere, and also nobody will get to know about it. So, everyone finds that it is an easy option.”* (ASHA, 16 years in practice, Jharkhand)

In addition to concerns about patient well-being, providers also expressed concerns about legal or professional liability for supporting clients who self-manage an abortion, such as when stating, “we don't want to take the blame”. One pharmacist believed they would be “jailed for 7 years” if they were found to have supported SMA. An ASHA shared that she tells people in the community not to tell her if they self-manage an abortion because she does not want to be perceived as responsible:

*“I will advise her to take medicines from the doctor only. I tell her ‘If you don’t take doctor’s advice and eat medicines on your own, if something happens to you, then don’t tell me later. You will be responsible for it yourself.’”* (ASHA, 14 years in practice, Jharkhand)

Provider perspectives were shaped by client experiences. A pharmacy worker explained how some clients who were unprepared for the symptoms of a medical abortion accused him of dispensing the wrong medicine because they were not expecting so much bleeding. One pharmacy worker explained that a client’s concern for undisclosed symptoms creates conflict:

*“If she's experiencing a lot of [bleeding], like there are many people [who say] ‘I took the medicine and am facing lots of problems. You should have told me about that’. They are totally in angry mood, ‘You gave me wrong medicine,’ they say. But that’s the reason [an abortion] why they want to use this medicine. But when they have the problem [heavy bleeding] then they come and fight with us and get angry.”* (Pharmacy worker, 5 years in practice, Jharkhand)

Provider concerns were also apparent in the ways they spoke about SMA. For example, several pharmacy workers and one ASHA used language that would distinguish the use of abortion medications outside of a facility as a different action than getting an abortion in a facility. For example, they used phrases like “get a miscarriage done” or would say to someone “you should not go for abortion you should use the pills”, when referring to SMA. Full quotes illustrating this distinction are shown in Table 2.

Despite these concerns and lack of support for SMA in premise, providers played a practical role in directly managing or supporting clients through the SMA process. Three areas were identified where some providers took steps: providing information on medication use and potential side effects; dispensing medications; and providing support during or after the abortion (i.e. managing pain or providing post-abortion contraceptive counselling). Although most pharmacy workers indicated that they required a prescription from an MBBS-qualified doctor before dispensing medications, some accepted an unofficial prescription from an informal local provider or no prescription at all. One pharmacy worker described dispensing medications and discussing the process to many clients who did not consult a doctor first.

*“We get many such cases where they don't go to the doctor and come to me twice and thrice, the same lady. So, later based on my knowledge I give them the medicine. I give the medicine and I nicely explain to them about the flow of bleeding or extent of blood loss or for how many days she will have the bleeding. I tell her about the dosage of the tablet as to when all it has to be taken, I tell them about all this.”* (Pharmacy worker, 5 years in practice, Bihar)

Informal local providers, ASHAs, and Anganwadi workers were the provider types who most often described providing support to the client. One informal local provider described discussing symptoms with the client, adding more abortion medication if needed, and managing pain:

**Table 2.** Quotes from providers that distinguish medication abortion outside of a facility from procedural abortion in a facility

Provider type	Quote
ASHA	“Then we tell the woman that she should try with the medicine, if it is successful, well and good, **but if the medicine does not work, then you will have to go in for an abortion** if you don't want to keep the baby.” *(12 years in practice, Jharkhand)*
Pharmacy worker	“We give the suggestion, **you should not go for abortion you should use the pills.**” *(2 years in practice, Jharkhand)*
Pharmacy worker	“I tell them that first you try to use the medicine if it happens from this then you don’t have to go for abortion. **If you get medicine, then eat it and if it happens with it then it is okay. Otherwise, last condition is to do abortion**.” *(20 years in practice, Bihar)*
Pharmacy worker	“I had a friend, he didn’t want to keep the child. He wanted a gap of 3 years between his 2 kids. **He is educated, so he got a miscarriage done**.” *(5 years in practice, Jharkhand)*
Pharmacy worker	“Out here the local provider here **cannot do abortion they will only suggest medicine**.” *(5 years in practice, Jharkhand)*

*“She will tell us the process, how she has taken, then I will ask her what the problem is, then she will tell me, whether it is the stomach cramps, or if some remains is still there, or lot of bleeding is happening, if lot of bleeding is going, we give them more tablet. If the stomach is in pain, it means there are still remains inside, so we will give her that tablet [abortion medication]. Then too if the pain is not going away, then we give her an injection [pain medicine].”* (Informal local provider, 7 years in practice, Jharkhand)

While some providers offered examples of facilitating SMA, most commonly, they stated they would refer the person seeking an abortion to someone else.

### Providers prefer to direct clients towards clinician-managed abortion over SMA

**Figure 1.**
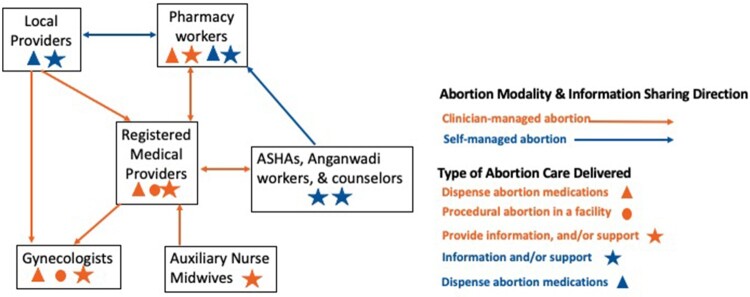
Abortion provider information sharing on pathways to self and clinician-managed abortion care, three states in India, 2022 This figure shows a map of information sharing on pathways to self and clinician-managed abortion care (clinician-managed in orange and self-managed in blue) mentioned by providers interviewed in this study or mentioned (i.e. gynaecologist) the pathway direction; and the type of care delivered by each provider type. Support includes managing medications, managing pain, and/or providing contraceptive counselling.**Definitions:** Pharmacy workers: pharmacists, chemists, and shop workers; LP: informal local providers; ASHA: accredited social health activists; counsellor: health counsellor; ANM: auxiliary nurse midwives; Bachelor of Medicine, Bachelor of Surgery (MBBS)-qualified doctors

While some providers gave information on pathways to SMA care, the primary and preferred pathways to care identified in this analysis centred around directing clients to clinician-managed care provided by MBBS-qualified doctors. All provider types indicated they provided information on MBBS-qualified doctors for a clinician-managed abortion routinely (ASHAs, Anganwadi workers, counsellors and auxiliary nurse midwives), to receive a prescription (pharmacy workers), and for abortion care at later stages of gestation (informal local providers). Figure 1 shows pathways to abortion care that participants discussed with clinician-managed activities shown in orange. This figure shows that providers of all types directed abortion seekers towards clinician-managed care. They played a role interfacing with health facility staff and accompanying the client through a clinician-managed abortion. ASHAs played both an authoritative role in the community as someone employed by the government stating, “I offer correct advice and tell the right thing”, as well as a compassionate parental figure saying their clients are “like a daughter to me”. One ASHA explained a typical abortion referral process to a facility:

*“They first contact ASHA and tell her that this is the situation. [I tell them] what I do and what I do not do, where to go and where not to go. I will tell them to go to [the facility in] Sadar first and I will also accompany them to Sadar. I will go and speak to the doctors and nurses over there and see what suggestion they give. If they say, first take this medicine, I give her the information. I am advising her. I take her to Sadar [to] get her abortion done over there by the nurse. Then I take advice from the doctor and tell her that this is what has happened. I have got her abortion done. Now she wants some [pain] medicine tablets. If needed, the doctor will write it down or else it is not needed.”* (ASHA, 14 years in practice, Bihar)

One Anganwadi worker explained the support she offers to refer clients and guide them through the clinician-managed abortion process:

*“If she says that she doesn't know where to go and whom to consult then I will go with her and tell her about the procedure. We will be with her and guide her like where to go and whom to consult. We stay with her and help her in all possible ways. If they come to us saying that they don't know how to proceed then I go along with her and take her to the hospital and we give all the possible help to her.”* (Anganwadi worker, 6 years in practice, Tamil Nadu)

Informal local providers indicated they provided information to abortion seekers on MBBS-qualified doctors if a pregnancy was at a later stage of gestation, while most auxiliary nurse midwives discussed referring to MBBS-qualified doctors for abortion at all stages and providing support for clinician-managed clients. MBBS-qualified doctors indicated they may also refer to a specialised gynaecologist for later stage abortion or tubal ligation.

### Providers share information on pathways to SMA-related care despite their concerns

Despite providers’ lack of support for SMA, participants acknowledge that SMA is occurring in the community and discussed pathways to SMA-related care (e.g. information, medications and/or support) with clients. A map of pathways to self and clinician-managed abortion care that participants in this study discussed is illustrated in Figure 1 with SMA-related activities shown in blue. This figure demonstrates that pharmacy workers and informal local providers provide information to people seeking SMA bidirectionally to access medications or to obtain unofficial prescriptions. Some pharmacy workers shared that “most of my customers are my acquaintances or refer by a local provider”. Another pharmacist reported dispensing medications to anyone and then said, “we advise them to consult a doctor before taking this”. This pharmacy worker said they refer clients to consult an MBBS-qualified doctor but will dispense abortion medications even if the client is unwilling so long as the client states that the pregnancy is in the early weeks of gestation. For later pregnancy durations, the pharmacy worker would not dispense medications and referred the client to an MBBS-qualified doctor or gynaecologist.

*“I tell them to meet the doctor because it may become a problem. If she doesn’t accept, then I give her the tablet. I ask how long she’s been pregnant for. If she’s more than 2 months, then I’ll ask her to go to the doctor.”* (Pharmacy worker, 5 years in practice, Bihar)

Informal local providers are a source of SMA care in communities who dispense medications and provide information and support. Like pharmacy workers, informal local providers will also advise clients with a more advanced pregnancy to see a MBBS-qualified doctor or gynaecologist to “get washed” [procedural abortion].

*“I take responsibility if pregnancy is 1-1.5 months long and if it is beyond that then I don't know what can happen in that case. I already told her that in such cases problems arise, like bleeding is there and they have many problems, so I ask them to get it washed.”* (Informal local provider, 10 years in practice, Bihar)

ASHAs and Anganwadi workers in this study indicated they typically do not directly assist with SMA care and advocate that clients seek clinician-managed abortion care. However, one ASHA described providing SMA care (information and support) if a client was unwilling to go to a facility:

*“If she is stubborn towards getting it [clinician-managed abortion] done, then I will help her. If she is asking me to help, I would help her, as I help them as a social worker. I would make her understand to not do it, but if she is stubborn, I will have to help her out.”* (ASHA, 17 years in practice, Bihar)

Auxiliary nurse midwives and MBBS-qualified doctors did not indicate that they play a direct role in supporting SMA care in the community. However, they stated that they provide post-abortion care such as confirmation of complete abortion or contraceptive counselling to clients presenting at facilities.

## Discussion

This study provides first-hand accounts of provider perspectives and roles in SMA in India and describes information on pathways to self- and clinician-managed abortion care. Most providers expressed *conditional* support for abortion care – only when abortion is sought for the “right reason”. Some of these “right reasons”, such as economic hardship, reiterated conditions where abortion is legal, stipulated by the MTP Act.^1^ Pharmacy workers, informal local providers, ASHAs, Anganwadi workers, and counsellors played a role in SMA care by fulfilling various tasks including describing usage and potential side effects, dispensing medication, and providing additional support during or after the abortion including managing pain or providing post-abortion contraceptive counselling. Auxiliary nurse midwives and MBBS-qualified doctors indicated that they did not provide direct care to SMA users in the community but that they instead provide post-abortion care in facilities.

Providers who do support, educate, and provide information to SMA clients are key intersection points between people who self-manage their abortions and the formal health system. Our findings show that while providers displayed opposition to SMA, they acknowledged that SMA is happening in the community, and that many SMA clients do interact with the formal health sector at varying points of the abortion process. This is consistent with previous work in India that has shown community health workers serve as both barriers and facilitators to access to abortion care.^21^ The practice of SMA has broadened the responsibility of delivering abortion care to actors outside of facilities; therefore, alignment between health facilities and providers who interact with SMA clients is needed to improve the quality of care delivered.^33,34^ However, this raises the important question of where the institutional mandate would come from, given the heterogeneity of the public/private abortion care networks and the lack of regulation around drug dispensing. Collaboration among actors within heath facilities and those serving people outside of facilities has the potential to leverage both community knowledge and institutional power to create a supportive and collaborative approach to service delivery. This synergy may be possible starting with incremental support for self-managed abortion by first expanding the use of telehealth for abortion^35^ to confer greater institutional support for remote provision of abortion with the goal of eventual increased acceptance of SMA by health system leaders and policy makers in the country. Notably, pursuing this integration is not only a matter of improving health metrics; it also speaks to upholding individual’s rights. Ensuring that safe abortion options are available in practice (and not just in premise) respects an individual’s autonomy in healthcare decisions and their right to access essential health services without undue hindrance.

Abortion stigma and health system constraints may limit the quality of care. Community health workers such as informal local providers, ASHAs, and Anganwadi workers are often a first point of contact between communities and the health system and are an important focus for understanding SMA and healthcare seeking in general.^21,36^ Our findings demonstrate that providers of all types are interacting with and supporting SMA users at various points; however, abortion stigma,^37^ some of which may be rooted in government policy (i.e. only endorsing abortion for the right reason), serves as a barrier to quality care. This selective endorsement, while reflecting the legal framework of the MTP Act, reveals how providers’ interpretations can reinforce abortion stigma by implicitly suggesting that only those meeting socially constructed criteria – such as socioeconomic status – are deemed “worthy” of care, thereby contributing to the broader social production of stigma as described by Kumar et al.^37^ Additionally, community health workers often operate under the constraints of limited institutional support, low pay, and the rigid hierarchical structure of the health system^38^ where abortion training and resources are directed at MBBS-qualified doctors. A robust body of literature on disrespect and abuse in childbirth demonstrates that gender inequities and unequal power dynamics in the health system undermine quality of care and enable a culture of social and professional inequity.^39,40^

Our findings show that pharmacy workers play an important role in both clinician- and self-managed abortion care given their role in describing usage, dispensing medications, and providing referrals for both facility and SMA clients. In India, the lack of regulatory oversight of pharmacies both enables greater accessibility of abortion medications but also complicates the assurance of quality standards and the provision of reliable information. Pharmacies and drug shops are often a preferred source of health care in low- and middle-income countries because of their convenience, privacy, anonymity, and low cost,^41^ and have been shown to be a source of medical abortion access in India.^42^ Our findings also demonstrate that pharmacy workers may confuse or obscure the abortive effects of medical abortions with the words they use such as by telling clients to “try the medication” and if it doesn’t work, then “have an abortion”. While terms such as menstrual regulation^43^ or “missed period pills”^44^ may be a potential strategy for garnering abortion support and expanding access, they can also unintentionally confuse clients and lead to a lack of preparation, misaligned expectations, and potentially inhibit quality care. Access to clear and accurate information is a critical component of high quality abortion care^45,46^ and is indicated as necessary in the WHO abortion care guidelines for SMA.^6^ Previous work on SMA has shown lower information provision in pharmacies when compared to facility-based care,^47^ and barriers to providing quality information from pharmacists include misconceptions and discomfort supplying abortion information.^16^ Pharmacies can serve as a key point of intervention to improve quality of care through clear and accurate language and supplying easy to understand information. However, operating within an unregulated policy environment – where pharmacies dispense abortion medications without prescriptions – poses significant challenges for policymakers in India, and highlights the crucial task of effectively expanding access to abortion care in the country.

Our findings of conditional support for abortion care as well as support for SMA in “practice” illustrate important considerations for health system leaders and policymakers to fully engage with WHO guidelines that support SMA. Understanding provider concerns as well as the work they do to support SMA – despite their concerns – helps illustrate an opportunity to align national policy more closely with global care standards by recognising and supporting what is occurring in practice at the local or organisational level. Previous work on post-abortion care in Senegal shows that when policy environments are not aligned with clinical practice, quality of care suffers, and system-level care metrics are inaccurate.^48^ This perpetuates the widely documented challenges of improving the quality of abortion data worldwide.^49^ Implementing WHO guidelines on SMA requires collaboration across various sectors including legal, educational, and medical communities, to create a supportive environment for providers to deliver evidence-based care that reduces barriers for patients and addresses system and organisational-level factors that perpetuate abortion stigma and hinder the delivery of quality care.

This study explores provider perceptions of SMA in India across a targeted sample, providing crucial insights into the informal support networks and the challenges faced by providers under the regulatory environment. By documenting these realities, our findings can inform policy revisions aimed at increasing access to safe abortion care. Additionally, understanding these provider perspectives can help tailor community-based interventions that improve the quality of care and reduce stigma, aligning with WHO recommendations for quality abortion care^6^ and incorporating a rights-based lens where policies explicitly aim to empower individuals with the information and means to manage their reproductive health.

This study built on previous work that has conducted pathway analysis^42^ and added an SMA information sharing component in a larger geographic area with more provider roles. This study also has limitations. First, our study does not extensively explore the economic motivations behind providers’ actions in facilitating SMA. Previous work suggests that provider actions may be driven by informal or formal income opportunities.^50^ Future research could benefit from directly addressing the role of provider economic incentives in SMA referral practices and care. Second, it is possible that the legal context of SMA in India may have impacted participants’ willingness to speak openly about their role in SMA care. Third, as a sub-analysis, the interviews were not specifically designed to capture all aspects of referral networks, which means that some pathways may have been missed. Finally, we acknowledge that our sample, though targeted, was not large enough to encompass the full diversity of provider experiences across different regions. However, this study was primarily exploratory, aimed at identifying initial patterns and insights into provider perceptions of SMA and can inform future, more extensive research.

## Conclusion

Despite provider concerns, SMA care and information on pathways to access SMA exist in India. Understanding the complex dynamics of provider perspectives, roles, and information sharing on pathways to care can inform improvements to comprehensive reproductive health policies and interventions to promote client-centred abortion care – including SMA – and address provider concerns. Community health workers are an important source of SMA support and should be empowered to address client needs. Pharmacies can serve as a key point of intervention for improving quality of care by bolstering the accuracy and clarity of information presented. There is a need for synergies between the formal health sector and SMA support networks to advance person experiences and reinforce quality abortion care as a human right.

## Supplementary Material

Supplemental Figure S2. Example interview guide

Supplemental_Figure_S1.docx

Supplemental Table S1: Qualitative Codebook

## Data Availability

The data that support the findings of this study are available from Ibis Reproductive Health upon reasonable request.

## Provenance statement

This article was not commissioned and went through external peer review.
