# Supplementary material for: Supporting self-managed abortion care in “practice not premise”: a qualitative study of provider perspectives, roles, and information pathways to care in India*
Source: Sex Reprod Health Matters. 2025 Jul 21;33(1):2531680. doi: 10.1080/26410397.2025.2531680 (PMC12351739; doi:10.1080/26410397.2025.2531680)
Supplement: Supplemental Figure S2. Example interview guide [file ZRHM_A_2531680_SM0066.docx]

**Supplemental Figure S2. Example interview guide**

**Understanding medication abortion self-use in India**

**In-depth interview guide: Community health worker (ASHA/ANM/Pharmacist/Local provider)**

[To be filled out prior to the interview]

Study ID:

Interviewer:

Date:

Has this participant given informed consent to be audio recorded? Yes No (check consent form, circle one)

Turn on voice recorder and check that it is working

Time recorder turned on: _________ am/pm

**If the interview is being conducted over the phone, ensure the participant has privacy and feels safe. Inform participant that the call is now being recorded.**

Say the participant’s **study ID number, today’s date, and time** so it’s on the audio recording.

Thank you for agreeing to speak with me. We’re interested in learning about how women in your community access sexual and reproductive health services, especially abortion. Your input will be helpful for understanding their experiences and your role in supporting access to these services.

Please keep in mind there are no right or wrong answers. Your answers will be kept anonymous and will not be linked to you. You may stop the interview at any time, and you do not have to answer any questions that you may feel uncomfortable answering. If you have any questions or need clarifications, you can feel free to stop me at any point during the interview.

*Note to interviewer: P – Pharmacist; LP – Local provider*

**I: Introduction**

1. Can you start by telling me a little bit about yourself and your work?
   1. Can you tell me about the work you do in the community? (Who do you serve/interact with? In what ways? About what topics?)
      1. ASHA: How long do you have to travel every day to carry out your work?
      2. ANM/P/LP: How long would a woman in your community need to travel to meet you?
      3. Why is your work necessary in the community?
      4. Who makes the decisions about what happens on a day-to-day basis in your area?
   2. What services do you provide/support that are related to sexual and reproductive health? (family planning, emergency contraception, abortion, sexually transmitted infections, HIV etc.)

**II: Perspective about abortion in the community:** Now, I would like to ask you some questions about how sexual and reproductive health and abortion is viewed by your community.

- 1. Can you tell me a little bit about your community?
     1. What does access to health care services look like in your community?
     2. Who provides health services in your community?
  2. When a woman wants to use family planning (like Mala D, nirodh, or IUD), what does the community think of her?
     1. Why do they think that?
     2. What do you think about this woman?
  3. When a woman tries to use emergency contraception (like iPill), what does the community think of her?
     1. Why do they think that?
     2. What do you think about this woman?

Now we would like to ask you what you know about how people in your community feel about ending a pregnancy.

- 1. Can you tell me more about how people in your community feel about ending a pregnancy?
     1. Are there different opinions?
     2. What are some of the reasons people in your community end a pregnancy?
        1. How do others in your community view those who end a pregnancy for this (these) reasons?
  2. What about other situations, such as unmarried women wanting to end a pregnancy, or a married couple not wanting a child hence ending a pregnancy?
     1. Does this happen in your community?
     2. How do others view those who end their pregnancy for these reasons?

**III. Decision-making:** Now, I’m going to ask you about how women make decisions about abortion.

1. Why do some women want to have an abortion?
   1. When/for what reasons do you think should women not be allowed to have an abortion?
   2. Do you think women may sometimes feel pressured to have an abortion?
      1. If yes, by whom or what (i.e., family members, community expectations/norms, etc.)?
   3. Do you think women already know how to get an abortion?
      - 1. What do they know?
        2. How do they know? *(from whom do they know)*
2. Do people approach you for help/advice about abortion?
   - 1. How often do people ask for help or advice about abortion? *(frequency in a month or in a week)*
     2. Who comes to you usually? *(the pregnant woman or someone else – their relationship to the pregnant woman)*
        - 1. If not the pregnant woman: Why do you think this person comes to you (and not the pregnant woman)?
          2. If pregnant woman: Does the pregnant woman come to you on her own or accompanied by others? *(Who accompanies her?)*
     3. How do they refer to abortion? *(What terms do they use to refer to abortion?)*
     4. When someone who is not the pregnant person approaches you for help/advice about abortion, what do you do? *(what is the partner/family member/friend’s journey like?)*
     5. Why do you think they come to you for help/advice? *(What do you think is your role – in decision-making? In accessing methods? Support/information? Follow up?)*
        1. Do they approach others before coming to you? Whom (all) do they approach?
        2. What do you do when you do not know how to advise them/how to help them?
     6. What methods do women use to end their pregnancy?
3. *(For each method the respondent shares)* How do they decide which method to use? Do they use more than one method?
4. *(For each method the respondent shares)* How do they access these methods? *(Who helps them access these methods?)*
   - 1. Do you think she would use the same abortion method the next time she wants to have an abortion? (*Why/why not?*)

**IV. Abortion journeys – I will now share some situations with you. Based on each situation, I will ask you some questions about how you have/will handle such cases.**

- - - 1. Seema is married and 24 years old. She has three children and is 6 weeks pregnant. She wants to have an abortion using pills. She is coming to you for help/advice. Can you think of some instances where this may have happened to you? (*encourage respondent to share examples*)
    1. What information/advice would you give Seema? *(how to make a decision about abortion/how to choose abortion method/where to access service or pills/what to expect/when to seek additional care/post-abortion family planning; what questions would you ask?)*
    2. Would you help Seema with getting abortion pills? (Note to interviewer: Respondents may say that they take them to an informal provider/other methods. Interviewer to ask about these other methods and then ask questions specific to MA pills)
       1. ASHA/ANM: How do you help Seema with getting the pills? Which pills would you advise her to take?
       2. P: Would you sell abortion pills to Seema? Which pills would you sell to her?
       3. Would you advise Seema about how to take the pills? What would you tell her?
       4. How would you decide how much you would charge her?
          1. Probe: What is the amount you would charge her?
       5. Would you help her in any other way? *(supporting during the abortion, information on what to expect, possible side effects, when to seek complications, others – for each aspect ask the respondent what they tell/do)*
    3. What do you think happens to Seema after she approaches you?
       - 1. Would you try to follow up with Seema to find out what happened with the pregnancy?
    4. Do you think Seema would want to get a family planning method after this experience?

1. Why or why not?
2. If yes: Do you know if Seema would use the family planning method? Why/why not?
   - 1. Do you think Seema would be open to having an abortion again in the future?
3. Why or why not?
   - - 1. *(Skip to 3 for LP)* Renu is unmarried and 19 years old. She is worried that people will find out she is pregnant, because she is unmarried. She refuses to go to a hospital or provider/doctor in her community because she is worried about being recognized. She comes to you for advice or help about getting an abortion without anyone else finding out. Can you think of some instances where this may have happened to you? *(encourage respondent to share some examples)*
4. What information/advice would you give Renu? *(where to access service or pills/what to expect/when to seek additional care/post-abortion family planning; what questions would you ask?)*
5. Why do you think Renu does not want to go to a doctor? *(Has anyone described poor quality care they received from a doctor? What made it poor quality?)*
6. Would you help Renu? How would you help?
   1. If Renu was an unmarried young woman who wanted to manage her abortion without a doctor, what would you do?
7. Do you think Renu would be open to having an abortion again in the future?
   1. Why or why not?
   2. Would Renu want to do it on her own or with the support of a doctor? Why?
      - 1. Vimala is 23 years old and married. She has three children. Even though she was using birth control pills, she is 5 weeks pregnant. She attempted to end her pregnancy on her own with pills, without going to a doctor. She is not sure if her abortion is complete. Can you think of instances where this may have happened to you? (*encourage respondent to share examples*)
8. How often does this happen? *(frequency in a month or in a week for pregnant person and someone else)*
9. What information/advice would you give Vimala? *(what questions would you ask? what pills did she take/what did she experience/whether her abortion is complete or not/post-abortion family planning)*
10. Would you help Vimala? How would you help?
11. Do you think Vimala would be open to having an abortion again in the future?
    1. Why or why not?
12. Would Vimala want to do it on her own or with the support of a doctor? Why?

**V. Improvements/recommendations**

- - - 1. Is there anything you think could improve the experiences of women undergoing abortion?

1. When you think of the clients who have had abortions, are there any health services you think could help them that they aren’t already getting?
2. What about those who are doing it without a doctor? How can their experience be improved?
   - - 1. Is there anything that could help you support women undergoing any type of abortion?
   1. What about those who are doing it without a doctor?
      - 1. If you provide a good service and community members have a good experience, who gets the credit? Are you appreciated by your supervisor/peers/superiors?

**VI. Closing**

Thank you so much for your time and willingness to participate. Those are all the questions I have for you today. Do you have any other questions about the interview?

**Turn off recorder.** Time turned off: ____________

**VII. Demographic data questions:**

- - - 1. How long have you been carrying out your current job? _______ years
      2. What is your highest educational level?
         1. Completed secondary/high school
         2. Completed undergraduate degree/vocational training
         3. Completed postgraduate degree
      3. What is your marital status?
         1. Married
         2. Single/separated/divorced/widowed
      4. Do you have children?
         1. Yes ______ (number of children)
         2. No
