# Supplementary figures and images for: Supporting self-managed abortion care in “practice not premise”: a qualitative study of provider perspectives, roles, and information pathways to care in India*
Source: Sex Reprod Health Matters. 2025 Jul 21;33(1):2531680. doi: 10.1080/26410397.2025.2531680 (PMC12351739; doi:10.1080/26410397.2025.2531680)

**Supplemental Figure S1.** Map of India, included states: Bihar, Jharkhand, and Tamil Nadu

**
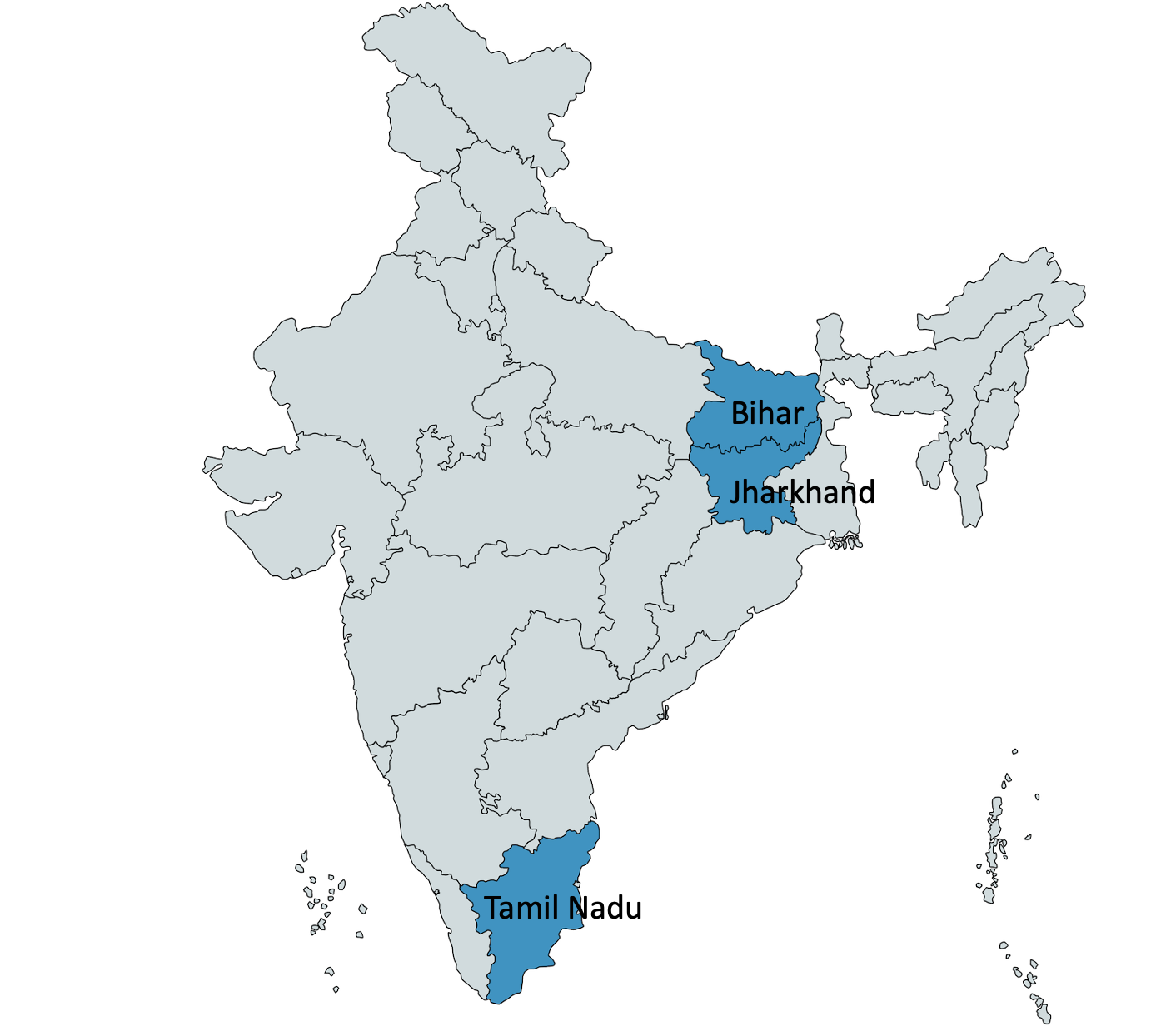
**

Supplement: Supplemental_Figure_S1.docx [file ZRHM_A_2531680_SM0065.docx]
